# Stress in Context: Morpho-Syntactic Properties Affect Lexical Stress Assignment in Reading Aloud

**DOI:** 10.3389/fpsyg.2016.00942

**Published:** 2016-06-22

**Authors:** Giacomo Spinelli, Simone Sulpizio, Silvia Primativo, Cristina Burani

**Affiliations:** ^1^Department of Psychology, University of Western Ontario, LondonON, Canada; ^2^Department of Psychology and Cognitive Science, University of TrentoTrento, Italy; ^3^Fondazione Marica De Vincenzi ONLUSTrento, Italy; ^4^Dementia Research Center, Department of Neurodegenerative Disease, University College LondonLondon, UK; ^5^Institute of Cognitive Sciences and Technologies (ISTC-CNR)Rome, Italy; ^6^Department of Life Sciences, University of TriesteTrieste, Italy

**Keywords:** stress assignment, reading aloud, grammatical category, morpho-syntactic context, stress neighborhood

## Abstract

Recent findings from English and Russian have shown that grammatical category plays a key role in stress assignment. In these languages, some grammatical categories have a typical stress pattern and this information is used by readers. However, whether readers are sensitive to smaller distributional differences and other morpho-syntactic properties (e.g., gender, number, person) remains unclear. We addressed this issue in word and non-word reading in Italian, a language in which: (1) nouns and verbs differ in the proportion of words with a dominant stress pattern; (2) information specified by words sharing morpho-syntactic properties may contrast with other sources of information, such as stress neighborhood. Both aspects were addressed in two experiments in which context words were used to induce the desired morpho-syntactic properties. Experiment 1 showed that the relatively different proportions of stress patterns between grammatical categories do not affect stress processing in word reading. In contrast, Experiment 2 showed that information specified by words sharing morpho-syntactic properties outweighs stress neighborhood in non-word reading. Thus, while general information specified by grammatical categories may not be used by Italian readers, stress neighbors with morpho-syntactic properties congruent with those of the target stimulus have a primary role in stress assignment. These results underscore the importance of expanding investigations of stress assignment beyond single words, as current models of single-word reading seem unable to account for our results.

## Introduction

Recent years have seen a growing interest in how readers assign lexical stress to both known and novel words. This is especially true for free-stress languages such as English or Italian, where stress is neither orthographically marked nor predictable based on rules. Among the factors found to affect stress assignment, three received special attention: lexical retrieval (i.e., the stress pattern stored within each word’s entry in the mental lexicon: Colombo, 1992; Rastle and Coltheart, 2000), stress dominance (i.e., the most frequent stress pattern in a language: Colombo, 1992; Rastle and Coltheart, 2000) and stress neighborhood, that is the proportion of words sharing the stress pattern and orthographic final sequence (e.g., most English disyllables ending in -*et* bear first-syllable stress: Burani and Arduino, 2004; Arciuli and Cupples, 2006; Ševa et al., 2009; Arciuli et al., 2010; Sulpizio et al., 2013; Burani et al., 2014; Jouravlev and Lupker, 2014). Recently, a few studies investigating reading in English and Russian (Arciuli and Cupples, 2006; Jouravlev and Lupker, 2014) also reported an effect of stress typicality (i.e., the dominant pattern within a grammatical category). They found that grammatical categories not only play a role in stress assignment, but also interact with stress neighborhood. For instance, in Russian no dominant stress pattern is distinguishable overall, however, adjectives show a clear preference toward trochaic stress. Using both a reading and a lexical decision task, Jouravlev and Lupker (2014) found facilitation for typically stressed Russian adjectives (i.e., trochaic). Conversely, atypically stressed adjectives (i.e., iambic) were disadvantaged, especially when stress neighborhood was inconsistent.

However, while reading, morpho-syntactic properties – such as gender, number, and person – other than grammatical category are active, and whether they play any role in the process of stress assignment is currently unknown. We used Italian to investigate such issue.

In Italian, the position of lexical stress is predictable from either orthography or phonology only in a small number of cases: when the last syllable is marked with a diacritic, it bears stress (orthographic rule, which occurs in ∼2% of words, e.g., poverTÀ ‘poverty’)^1^; when there is no diacritic on the last syllable and the penultimate syllable ends with a consonant, the latter bears stress (phonological rule, e.g., *conCERto* ‘concert’)^2^. For the most part of the Italian lexicon, however, the latter rule does not apply. Thus, readers mainly use lexical retrieval and distributional information such as stress dominance and stress neighborhood in order to assign stress. Note that unlike Russian, Italian shows an asymmetrical distribution of stress patterns overall, with about 80% of words bearing penultimate stress (e.g., *teSOro* ‘treasure’) and 18% bearing antepenultimate stress (e.g., *Anima* ‘soul’: Thornton et al., 1997). Accordingly, Colombo (1992) argued for a primary role of stress dominance in Italian, with penultimate stress working as a default. More recent studies, however, succeeded in finding an effect of stress dominance only in populations with little or reduced ability to detect more specific distributional information in the lexicon. Examples of such populations are second graders, children with dyslexia and adults with acquired language disturbances (for a review, see Sulpizio et al., 2015). On the other hand, stress neighborhood, a more specific source of distributional information, has been reported as the strongest factor driving stress processing and stress assignment in both low-frequency words and non-words for unimpaired adults and older children (Sulpizio et al., 2013; Burani et al., 2014), at least in reading aloud (Burani and Arduino, 2004; Colombo and Sulpizio, 2015).

While the literature focused on the relative role of stress dominance and stress neighborhood in Italian readers, no attention was paid to the role of morpho-syntactic properties such as grammatical category, number, gender, and person, in stress processing. These properties are known to affect word processing in different tasks and languages (Bates et al., 1996; Federmeier et al., 2000; Bentrovato et al., 2003; Monaghan et al., 2005; Vigliocco et al., 2008; Arciuli et al., 2012; see also Lalović, 2010).

The distribution of stress patterns with respect to morpho-syntactic properties raises two noteworthy questions. First, do Italian readers, like English speaking and Russian ones, use distributional information specified by grammatical category when assigning stress? While all grammatical categories in Italian show a prevalence of penultimate stress words, there are some outstanding differences between them, with adverbs and nouns showing higher proportions of dominant stress words than adjectives and verbs (95.3; 84.9; 76.2, and 70.3%, respectively). It may be the case that readers are sensitive to those differences. Second, how specific is stress neighborhood information? Note that stress neighborhood is defined as the number of words sharing the same final sequence (i.e., the last three graphemes in Italian) and stress pattern. For instance, stress neighborhood for the final sequence *-era* is prevalently penultimate, as most words ending in *-era* have penultimate stress (e.g., *panTEra* ‘panther’). However, words with the same final sequence can have different stress patterns depending on their morpho-syntactic properties. In fact, third person verbs ending in *-era* mostly bear antepenultimate, rather than penultimate, stress (e.g., *MOdera* ‘he/she moderates’). Thus, in reading, morpho-syntactic properties may interact with stress neighborhood in specifying a stress pattern. Here, we address those issues by presenting data coming from both word and non-word reading.

Experiment 1 investigated the role played by grammatical category in reading words aloud. Studies addressing stress processing in reading aloud showed that there is no advantage for penultimate stress words (Burani and Arduino, 2004; Burani et al., 2014), although most Italian words bear penultimate stress. However, since nouns were generally used and verbs show a lower proportion of penultimate stress words than nouns, we might expect this difference to be reflected in readers’ performance, especially when morpho-syntactic processing is enhanced. We were also interested in the role of morpho-syntactic properties in stress assignment to novel words. Thus, Experiment 2 turned to non-word reading aloud. Non-words were constructed in such a way that information coming from stress neighborhood and information specified by morpho-syntactic properties (i.e., grammatical category, gender, number, or person) contrasted with each other. Our question was whether morpho-syntactic properties could have a stronger influence than stress neighborhood in driving participants’ responses.

## Experiment 1

In this experiment, we aimed to assess the extent to which Italian readers use grammatical category as a source of distributional information in order to assign stress. In Italian, stress patterns are not distributed equally within grammatical categories. This fact might have been underestimated so far because previous analyses (Thornton et al., 1997) were based on lemmas (i.e., the canonical or citation form of a word) rather than word forms (i.e., any inflected form of a word). However, in Italian, counts made on lemmas may differ dramatically from word forms. This is especially true for verbs, which show different inflectional markers for each of the 12 simple tenses, each of the three conjugations, and each person. Most importantly, they show alternating stress patterns and number of syllables within inflectional paradigms. Thus, we analyzed word forms in *phonItalia* (Goslin et al., 2014), a lexical database providing orthographic and phonological information for 120,000 word forms extracted from *CoLFIS* (Bertinetto et al., 2005). The results showed that, although penultimate stress is the dominant pattern for each grammatical category, verbs have a lower proportion of penultimate stress word forms as compared to nouns (70.3 vs. 84.9%).

That proportion for verbs may be even lower depending on what one considers to be the relevant source of distributional information. In languages such as Italian and English, morphology is known to affect the position of stress, as suffixes are either stressed or unstressed (Rastle and Coltheart, 2000). In Italian, however, the first, second, third, and sixth persons in present indicative and subjunctive may have either ultimate, penultimate, antepenultimate, or even pre-antepenultimate stress (sixth person only, e.g., *GIUdicano* ‘[they] judge’). In those verb forms, the position of stress is unpredictable, provided that the penultimate syllable does not end with a consonant. Thus, one may want to consider only those forms where stress is not fixed, i.e., governed by neither inflectional suffixes nor stress rules. We selected those verb forms from *phonItalia* and found that penultimate stress patterns dropped to 60.7%, with antepenultimate stress patterns accounting for 39.3% of the set. Furthermore, four-syllable and five-syllable verb forms turned out to prefer antepenultimate rather than penultimate stress.

In sum, there is evidence that the proportion of penultimate and antepenultimate stress patterns differs between nouns and verbs, with the latter showing a relatively larger proportion of antepenultimate stress words. We wanted to test whether those distributional differences are sufficient to replicate, in Italian, the findings reported by Arciuli and Cupples (2006) for English and Jouravlev and Lupker (2014) for Russian. Note that, although penultimate stress is the dominant pattern for both nouns and verbs, we might expect a different pattern of results for the two grammatical categories if such distributional information is relevant to Italian readers when assigning stress. Besides, differences, if any, might be larger when morpho-syntactic processing is enhanced, i.e., when information concerning the grammatical category of the word is available to readers beforehand.

We therefore designed a reading aloud task where nouns and verbs were presented either isolated or preceded by a congruent context word (i.e., an article in the case of nouns, and a pronoun in the case of verbs). In line with previous studies where stress neighborhood was controlled for, we did not expect processing times differences between penultimate and antepenultimate stress nouns. In contrast, we hypothesized that if Italian readers are sensitive to distributional differences at the level of grammatical categories, then the larger proportion of antepenultimate stress words within verbs may lead to some processing advantage for antepenultimate over penultimate stress for verbs. A larger effect was hypothesized for the with-context as compared to the without-context condition.

### Participants

Thirty-two undergraduate students (11 males, mean age: 24.2, *SD*: 1.9) from Sapienza University of Rome took part in the experiment. They were all native Italian speakers with normal or corrected-to-normal vision. This study was carried out in accordance with the recommendations of the Research Ethics Committee of the Institute of Cognitive Sciences and Technologies in Rome, with written informed consent from all participants in accordance with the Declaration of Helsinki.

### Materials

We used low-frequency words in line with the assumption that distributional effects are more likely to emerge with low- rather than high-frequency words (Colombo, 1992). One-hundred and sixty low-frequency words, half nouns and half verbs, were selected from *phonItalia* (Goslin et al., 2014). All words had a final sequence with a VCV (vowel-consonant-vowel) orthographic structure. For each grammatical category, half of the words had a penultimate stress neighborhood, and half had an antepenultimate stress neighborhood. For each stress neighborhood, half of the words had a penultimate stress pattern, and half had an antepenultimate stress pattern. As a result, half words were consistent and half were inconsistent with their stress neighborhood. In sum, there were eight sets of 20 words each varying in grammatical category, stress neighborhood, and stress pattern. Words were chosen in such a way to cover as many morpho-syntactic combinations as possible. Nouns were either masculine or feminine, and either singular or plural. Verbs included first, second, and third person present indicative. Morpho-syntactic properties were matched across sets of the same grammatical category. Each set included 6–8 different final sequences. Of these, 2–4 final sequences were set-exclusive, and the other 4–5 final sequences appeared in another set. Each shared final sequence appeared half of the time in stress-consistent and half of the time in stress-inconsistent words. For each set, two final sequences were shared within grammatical categories (e.g., buF*Ere* ‘storms,’ MASch*ere* ‘masks’), and 2–3 between grammatical categories (e.g., FORf*ora* ‘dandruff,’ imPL*Ora* ‘[he/she] implores’). All words had three syllables, except for eight four-syllable words which were equally distributed across sets. Since the aim of the study was to compare responses to nouns and verbs with penultimate and antepenultimate stress, only sets differing in stress pattern were matched on initial phoneme and other variables like frequency, length in letters, bigram frequency and orthographic neighborhood (see **Table 1**). Due to the low-frequency of stimuli, familiarity ratings were collected from an independent group of participants who did not take part in the experiment. Twenty-one university students (12 females, mean age: 24.89, *SD*: 3.89) were asked to estimate the familiarity of all nouns and verbs on a seven-point scale, following the procedure described by Bates et al. (2001). Familiarity resulted not to be fully matched across the experimental sets of stimuli; thus, such measure was introduced as covariate in the analyses.

**Table 1:** Mean characteristics of stimuli in Experiment 1.

	Noun				Verb			
Variable	Consistent neighborhood		Inconsistent neighborhood		Consistent neighborhood		Inconsistent neighborhood	
	Penult	Antepenult	Penult	Antepenult	Penult	Antepenult	Penult	Antepenult
Frequency	7.40	8.10	7.40	8.10	3.20	3.40	4.80	5.05
Length	7.05	7.20	7.20	7.00	7.00	7.15	6.95	6.85
Bigram frequency	11.81	11.73	11.71	11.62	11.54	11.59	11.60	11.68
Orthographic complexity	0.60	0.75	0.90	0.75	0.60	0.65	0.55	0.55
*N* size	2.45	2.40	1.80	2.60	2.85	2.70	2.80	3.05
*N* frequency	8.53	8.65	7.09	7.67	13.40	11.54	8.33	6.67
*Familiarity*	6.37	6.12	6.60	6.00	6.29	6.18	5.92	6.30

*Penult = penultimate stress pattern; *Antepenult* = antepenultimate stress pattern. All the reported values are mean values. *Length* is in number of letters. *Bigram frequency* is log transformed on the basis of the natural logarithm. *Orthographic complexity* is based on the number of *c, g, sc*, and *gl* letters that are present in a given word (In Italian, these letters and letter clusters require contextual rules in order to be assigned the correct pronunciation; see Burani et al., 2006). *N size* indicates the number of neighbors that are obtained by changing the target’s letters one at a time. *N frequency* is the summed neighbors’ frequency (Wagenmakers and Raaijmakers, 2006). *Familiarity* is obtained from seven-point subjective ratings.*

Eighty filler words were also included. Half were nouns and half verbs, and for each grammatical category, half bore stress on the penultimate and half on the antepenultimate syllable. Filler words differed from experimental items in the following: (1) their stress neighborhoods were ambivalent (see Colombo et al., 2014), and thus, did not provide clear cues for stress; (2) they included a high proportion of four-syllable words (34 out of 80, 42.5%).

### Procedure

The list of 240 items was presented in four blocks, each composed of the same number of nouns and verbs, penultimate and antepenultimate stress neighborhoods, and consistent and inconsistent words. Effort was made to limit repetitions of the same final sequences within each block. The presentation order of stimuli within each block was randomized, and block order counterbalanced across participants.

Sentence context was also manipulated. Morpho-syntactic properties may be stored in the lexical entry of each word in the lexicon (e.g., Levelt et al., 1999). Nevertheless, words have been shown to benefit from a morpho-syntactically congruent context in lexical decision (Goodman et al., 1981; Seidenberg et al., 1984; Wright and Garrett, 1984; see also Carello et al., 1988 and works cited) and naming tasks (Bowey, 1996; Bentrovato et al., 2003; Lalović, 2010). Thus, in order to enhance the processing of the morpho-syntactic properties of words, we used a sentence context: words were read in two conditions, namely with context and without context. In the with-context condition, a fixation cross was displayed for 400 ms at the beginning of each trial. Then, the fixation cross disappeared and a context word appeared, with the first letter in the same spatial position of the fixation cross. After 425 ms, the stimulus was displayed next to the context word. Participants were asked to read aloud the stimulus (but not the context word) as quickly and as accurately as possible. Both the context word and the stimulus disappeared at pronunciation or after 1,000 ms since the stimulus onset. There was an interstimulus interval of 2,500 ms. In this condition, both the fixation cross and the context word were displayed slightly to the left of the screen, in order for the stimulus to appear in roughly the same position as in the without-context condition. The context word consisted of either an inflected article (e.g., *il* ‘the, masculine singular’) or a personal pronoun (e.g., *egli* ‘he’). All context words were morpho-syntactically congruent with their associated stimuli. The without-context condition differed from the with-context condition in that there was no context word, and both the fixation cross and the stimulus were centered on the screen.

Each participant read each stimulus once in either the with- or without-context condition. Of the four blocks, one pair of consecutive blocks was read in the with-context condition and the other pair was read in the without-context condition. Condition order was counterbalanced across participants. A practice block of 10 trials preceded each pair of blocks. The experiment was run in a quiet room using DMDX software (Forster and Forster, 2003).

### Results

Reaction times (RTs) were measured by means of a voice-key, and defined as the time difference between the appearance of the target stimulus on the screen and the onset of participants’ response. Response waveforms were then manually inspected with CheckVocal (Protopapas, 2007) in order to determine the accuracy and placement of timing marks. Invalid trials due to technical failures, as well as responses that exceeded the time limit, accounted for 1.4% of the data points and were discarded from the analyses. Phonemic and stress errors were also discarded, as they accounted for only 3.5% of the data points. Mean RTs are shown in **Table 2**.

**Table 2:** Mean latencies (and standard deviations) in ms for correct responses in Experiment 1.

	Consistent				Inconsistent			
	Without context		With context		Without context		With context	
Grammatical category	Penult	Antepenult	Penult	Antepenult	Penult	Antepenult	Penult	Antepenult
Noun	576 (76)	595 (77)	567 (88)	572 (84)	593 (68)	594 (80)	563 (88)	573 (79)
Verb	578 (75)	579 (88)	569 (86)	562 (85)	587 (85)	581 (91)	570 (84)	563 (83)

*Penult = penultimate stress neighborhood; *Antepenult* = antepenultimate stress neighborhood.*

Reaction times were analyzed using linear mixed effects modeling (Baayen, 2008) with crossed random effects for participants and items. The models were fitted with lme4 1.0-5 (Bates et al., 2013), and lmerTest (Kuznetsova et al., 2013) packages implemented in R 3.0.2 (2013–09–25, R Core Team, 2013). Models included near-maximal random structure to the extent allowed by convergence constraints (Barr et al., 2013). Once the models were fitted, outliers were identified and removed (employing 2.5 SD of the residual errors as criterion; Baayen, 2008). Statistics of the refitted models are reported. As materials were designed to compare penultimate and antepenultimate stress words within each grammatical category, verbs and nouns were analyzed separately; for each category, the same analyses with morpho-syntactic context (present vs. absent), word stress (penultimate vs. antepenultimate), and stress neighborhood consistency (consistent vs. inconsistent) as predictors were performed; over and above the variables of interest, familiarity was included as a covariate.

#### Nouns

Main effects of consistency (β = 23.27, *SE* = 11.06, *t* = 2.10, *p* = 0.03) and familiarity (β = -22.34, *SE* = 6.20, *t* = -3.60, *p* < 0.001) were found. The interaction between morpho-syntactic context and stress neighborhood consistency was significant (β = -21.08, *SE* = 7.75, *t* = -2.72, *p* = 0.006). Moreover, the three-way interaction was also significant (β = 24.84, *SE* = 11.00, *t* = 2.25, *p* = 0.02). No further effect reached significance (for all other effects: |1.15| < ts < |1.66|, ps > 0.1).

The three-way interaction was further investigated by splitting the data for word stress. When considering the antepenultimate-stress words, other than the effect of familiarity (β = -20.39, *SE* = 6.58, *t* = -3.09, *p* = 0.003), only the main effect of morpho-syntactic context was apparent (β = -23.43, *SE* = 7.66, *t* = -3.05, *p* = 0.004). Neither the effect of stress neighborhood consistency nor the stress neighborhood consistency × morpho-syntactic context interaction were significant (both *t*_s_ < 1). When considering penultimate-stress words, other than the effect of familiarity (β = -24.04, *SE* = 12.25, *t* = -1.96, *p* = 0.05), no effect reached significance (consistency: *t* = 1.83, *p* > 0.05; morpho-syntactic context: *t* = -1.29, *p* > 0.2). However, the stress neighborhood consistency × morpho-syntactic context was significant (β = -20.90, *SE* = 7.66, *t* = -2.72, *p* = 0.006), showing that the effect of context was larger for words with inconsistent (β = -31.83, *SE* = 5.8, *t* = -5.58, *p* < 0.001) than with consistent stress neighborhood (β = -10.98, *SE* = 5.32, *t* = -2.06, *p* = 0.03).

#### Verbs

The interaction between morpho-syntactic context and stress neighborhood consistency was significant (β = -15.54, *SE* = 8.06, *t* = -1.92, *p* = 0.05), with the morpho-syntactic context exerting a larger effect on words with inconsistent (β = -22.44, *SE* = 7.58, *t* = -2.95, *p* = 0.005) than with consistent stress neighborhood (β = -13.12, *SE* = 6.08, *t* = -2.15, *p* = 0.03). Also familiarity reached significance (β = -33.25, *SE* = 5.31, *t* = -6.25, *p* < 0.001). No further effect reached significance (|0.50| < ts < |1.07|, *p*s > 0.2).

### Discussion

Although stress patterns do not distribute equally in grammatical categories in Italian, we found no evidence supporting the claim that readers use this information when assigning stress. In line with previous studies using nouns (Burani and Arduino, 2004; Burani et al., 2014), we reported no significant differences between penultimate and antepenultimate stress nouns when stress neighborhood was controlled, and the use of context did not change such pattern. For the first time, we showed that the same is true for verbs. In Italian, verbs have a higher proportion of antepenultimate stress words than nouns, and this is especially true for the verb forms we used (i.e., first, second, and third person present indicative). Even in this case, however, no difference between penultimate and antepenultimate stress verbs was observed, regardless of the presence of context. Therefore, these results indicate no role for grammatical information in stress processing for adult Italian readers, possibly because in Italian differences between categories are not as large as in languages such as English and Russian.

As expected, we did find an effect of experimental condition: words were read faster in the with- than in the without-context condition. This effect, however, was significant only for inconsistent words, an interesting finding that will be discussed in Section “General Discussion.”

## Experiment 2

While adult Italian readers are insensitive to general distributional information (i.e., stress dominance in the language and within a given grammatical category), they rely on specific information (i.e., stress neighborhood) in order to assign stress to both words and non-words (Burani and Arduino, 2004; Sulpizio et al., 2013; Burani et al., 2014). However, previous studies do not say much about whether and how stress neighborhood is grammatically specific. In fact, most of the final sequences that have been used so far show a consistent proportion of either penultimate or antepenultimate stress words across all grammatical categories. For example, -*ola* has an antepenultimate stress neighborhood, and most feminine singular nouns, feminine singular adjectives, and third person present indicative verbs ending in -*ola* bear antepenultimate stress. Thus, it is not clear whether words and non-words are assigned stress on the basis of the overall count of their stress neighbors, or on the basis of just those neighbors with which they share morpho-syntactic properties.

Non-words are especially interesting in this respect. Unlike words, processing of morpho-syntactic information for non-words must be induced by using inflectional affixes and/or contextual information. Thus, on the one hand, morpho-syntactic processing may only be partial in reading tasks that involve isolated non-words (i.e., not primed by a context word). On the other hand, the role of morpho-syntactic properties in stress processing is more easily controlled with non-words than with words when a context word is used, because neither morpho-syntactic properties nor stress patterns can be retrieved from the lexicon. That is, all the relevant information to process morpho-syntactic properties and assign stress to a non-word must come from the written stimulus and contextual information. At the same time, it is important to note that non-word and word reading are tightly related. For example, dual-route models of reading assume that each input (word or non-word) activates both lexical and sub-lexical processing, and the outputs of the two routes interact in the phonemic buffer (Coltheart et al., 2001; Perry et al., 2010). Connectionist models show an even stronger picture, as each input the system receives is processed by the same mechanisms (e.g., Plaut, 1999; Harm and Seidenberg, 2004). Indeed, lexical effects on non-word reading have been systematically reported (see, e.g., Coltheart et al., 1977; Laxon et al., 1992; Peereman and Content, 1995), and there is evidence that such effects extend to non-word stress assignment (Arciuli and Cupples, 2006, 2007; Protopapas et al., 2007). In sum, although non-words are markedly different from real words, they can inform our view of the mechanisms involved in stress assignment and reading in general.

We thus used a non-word reading aloud task to assess the role of morpho-syntactic properties in stress assignment. Unlike previous studies, only inconsistent final sequences were used. In those final sequences, the stress pattern specified by stress neighborhood as a whole conflicts with the stress pattern specified by at least one subset of stress neighbors with shared morpho-syntactic properties, i.e., words that have both the same orthographic ending and the same morpho-syntactic properties. Note that shared morpho-syntactic properties include grammatical category as well as gender and number (for nouns), and person (for verbs). For example, -*ine* has a penultimate stress neighborhood, but masculine singular nouns ending in -*ine* bear antepenultimate stress most of the time. Our prediction was that, when no context is available, morpho-syntactic processing is reduced and readers assign stress on the basis of the non-words’ stress neighborhood. When context is available, however, morpho-syntactic processing is enhanced, and readers may assign stress basing on the non-words’ morpho-syntactically congruent stress neighbors, even though they conflict with overall stress neighborhood.

### Participants

Thirty-two undergraduate students (ten males, mean age: 23.8, *SD*: 2.8) from Sapienza University of Rome participated in the experiment. They were all native Italian speakers, with normal or corrected-to-normal vision. This study was carried out in accordance with the recommendations of the Research Ethics Committee of the Institute of Cognitive Sciences and Technologies in Rome, with written informed consent from all participants in accordance with the Declaration of Helsinki.

### Materials

We selected eight final sequences for which there was at least one subset of stress neighbors with shared morpho-syntactic properties that specified a different stress pattern from the one specified by the overall stress neighborhood. Half (-*era*, -*eri*, -*ine*, -*ita*) had a penultimate stress neighborhood and an antepenultimate stress subset, and half (-*ano*, -*ere*, -*ici*, -*ide*) had an antepenultimate stress neighborhood and a penultimate stress subset. Half had a noun subset (-*ano*, -*ere*, -*ici*, -*ine*) and half a verb subset (-*era*, -*eri*, -*ide*, -*ita*). For each final sequence, 16 three-syllable non-words were constructed, for a total of 128 stimuli. Orthographic consonant-vowel structures were proportioned to data from *phonItalia* (Goslin et al., 2014), with more non-words constructed for the most representative structures of each final sequence. Non-words had no orthographic neighbors. Other matching variables for the stimuli are shown in **Table 3**.

**Table 3:** Mean characteristics of stimuli in Experiment 2.

Variable	Stress neighborhood	
	Penult	Antepenult
Length	6.77	6.72
Bigram frequency	11.65	11.68
Orthographic complexity	0.63	0.63
*N* size	0.00	0.00

*Penult = penultimate stress neighborhood; *Antepenult* = antepenultimate stress neighborhood. All the reported values are mean values.*

One hundred and twelve filler non-words were also included. Filler non-words differed from experimental materials in that they included consistent final sequences (i.e., stress neighborhoods and subsets provided compatible information).

All materials were divided into two sets according to the grammatical category that was induced in the with-context condition (see below): (1) non-words presented as nouns, and (2) non-words presented as verbs. Within each set, half of the non-words had a penultimate stress neighborhood and half had an antepenultimate stress neighborhood.

### Procedure

Stimuli with the same final sequences were equally distributed in four blocks of 60 trials each. Thus, each block had the same proportion of penultimate and antepenultimate stress neighborhoods, and items presented as nouns and verbs in the with-context condition. The presentation order of trials within each block was randomized.

The procedure was the same as in Experiment 1, with half of the stimuli presented in the with-context and half presented in the without-context condition. Context words in the with-context condition were chosen so as to activate the morpho-syntactic properties of the intended subsets, and were either inflected articles or pronouns. For example, a non-word ending in -*ita* was presented as a third person present indicative verb when it was preceded by the third person pronoun *egli* ‘he.’ Unlike Experiment 1, however, participants were not given particular instructions concerning speed and accuracy, and there was no time limit for the response. Additionally, in the with-context condition, the context word and the stimulus appeared together, and participants were instructed to read aloud both. The main reason for these changes was to elicit morpho-syntactic processing that was as deep as possible. To validate the procedure used in Experiment 1, we ran a pilot study using timed trials on 12 participants. At the end of the timed experiment, participants reported that context words were occasionally ignored, and indeed, an effect of context could be found only for a portion of the results. These considerations suggest that a timed reading task may not be suited to the investigated effect. Thus, we decided to let participants read the stimuli with no time pressure. We also asked them to read context words aloud in order to make sure those words were fully processed and thus had an influence on the processing of the target stimulus.

As in Experiment 1, each participant read each stimulus once in either the with- or without-context condition, and condition and block order were counterbalanced. A practice block of 10 trials preceded each pair of blocks. The experiment was run in a quiet room using E-prime software (Psychology Software Tools, Pittsburgh, PA, USA)^3^. The experimenter coded each response as bearing either penultimate or antepenultimate stress. Phonemic errors were also noted.

### Results

**FIGURE 1:**
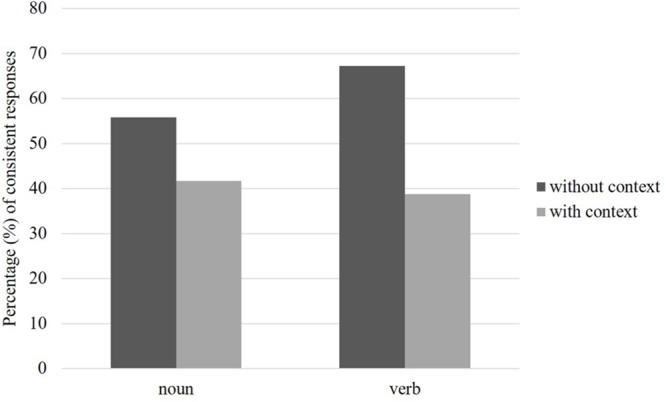
**Percentages of consistent responses for non-words presented as either nouns or verbs in the two experimental conditions, Experiment 2.** A response was classified as consistent when the non-word was assigned the same stress pattern specified by its stress neighborhood. Non-words were presented as either nouns or verbs in the with-context condition only. In the without-context condition, non-words were presented isolated.

Phonemic errors, accounting for 1.2% of the data points, were discarded from the analyses. Responses were classified as being either consistent or inconsistent with stress neighborhood. Percentages of consistent responses for non-words presented as nouns and verbs are shown in **Figure 1**. We used a mixed logistic regression analysis with crossed random effects for participants and items; morpho-syntactic context (presence vs. absence), induced grammatical category (noun vs. verb), and stress neighborhood (penultimate vs. antepenultimate) were entered as fixed factors and the consistency of responses as the dependent variable^4^. The logistic regression model was fitted using the *glmer* function in R software (version 3.0.2). Models included near-maximal random structure to the extent allowed by convergence constraints (Barr et al., 2013); the final model included random intercept for participants and items.

There was a main effect of induced grammatical category (β = 0.70, *SE* = 0.23, *z* = 3.01, *p* = 0.002) and a two-way interaction between morpho-syntactic context and induced grammatical category (β = -0.87, *SE* = 0.22, *z* = -3.83, *p* < 0.001). More importantly, the three-way interaction was significant (β = 1.25, *SE* = 0.32, *z* = 3.8, *p* < 0.001). No further effect reached significance (stress neighborhood: *z* = 1.8, *p* = 0.06; stress neighborhood × context: *z* = 1.3, *p* > 0.1; stress neighborhood × grammatical category: *z* < 1). The three-way interaction was further investigated by splitting the data for grammatical category. When stimuli presented as nouns were considered, both the main effects of stress neighborhood (β = 0.44, *SE* = 0.23, *z* = 1.8, *p* = 0.05) and morpho-syntactic context were significant (β = -0.88, *SE* = 0.19, *z* = -4.51, *p* < 0.001), but not their interaction (*z =* 1.37, *p* > 0.1). On the other hand, in the case of stimuli presented as verbs, both the main effects (morpho-syntactic context: β = -1.84, *SE* = 0.12, *z* = -14.67, *p* < 0.001; stress neighborhood: β = 0.72, *SE* = 0.26, *z* = 2.74, *p* = 0.005) and the interaction (β = 1.64, *SE* = 0.25, *z* = 6.54, *p* < 0.001) were significant, with the latter revealing that the morpho-syntactic context had an effect only for stimuli with penultimate stress neighborhood (β = 2.03, *SE* = 0.13, *z* = -14.83, *p* < 0.001), but not for stimuli with antepenultimate stress neighborhood (*z* < 1, *p* > 0.3).

### Discussion

The main results of Experiment 2 confirmed our predictions. In the without-context condition, most responses were driven by stress neighborhood, in line with previous results (Sulpizio et al., 2013). In the with-context condition, however, responses were inconsistent with stress neighborhood as calculated on all stress neighbors. Thus, when morpho-syntactic processing is possible, Italian readers use information coming from the subset of stress neighbors with the same morpho-syntactic properties as the stimulus. This claim is strengthened by the finding that when the two sources of information are in contrast, information coming from morpho-syntactically similar stress neighbors has a stronger influence than overall stress neighborhood information.

We also found that the effect of context, although holding for both induced grammatical categories, was stronger for non-words presented as verbs than for those presented as nouns. Moreover, the context interacted with stress neighborhood only for non-words presented as verbs, as only those with a penultimate stress neighborhood showed an effect of context. Note, however, that since the focus of this experiment was on morpho-syntactic properties rather than grammatical categories *per se*, stress neighborhoods and grammatical categories were not perfectly balanced in our materials. In fact, three penultimate stress neighborhoods out of four appeared in stimuli presented as verbs, and three antepenultimate stress neighborhoods out of four appeared in stimuli presented as nouns in the with-context condition. Thus, the reported findings may suggest that when stress neighborhood provides contrasting information, non-words presented as verbs are relatively more prone to be assigned antepenultimate stress as compared to non-words presented as nouns, with the latter showing a weaker tendency to be assigned penultimate stress. This finding would be consistent with the hypothesis tested in Experiment 1 (i.e., a difference between nouns and verbs due to the general stress pattern distribution). However, as this experiment was not designed to investigate the role of general distributional information in non-word stress assignment, the issue needs further scrutiny and is left for future research.

## General Discussion

The experiments reported in the present paper addressed the role of morpho-syntactic properties in assigning stress to both words and non-words in Italian. In Experiment 1, we found no evidence in favor of the view that the relatively different proportions of penultimate and antepenultimate stressed words across different grammatical categories may drive the performance of readers. In contrast, Experiment 2 showed that stress assignment to non-words is best described as the effect of information coming from the stress neighbors that share morpho-syntactic properties with the target non-word that is presented in a given grammatical context, rather than information coming from the total stress neighborhood.

To date, no study has addressed morpho-syntactic properties as a potential source of information for stress assignment in Italian. The present paper reports findings suggesting that they do play a role, but only at a very specific level. At the general level, the differences in the distribution of stress patterns across grammatical categories are not reflected in processing differences in word reading aloud, as shown by Experiment 1. Although our experimental manipulation may have not been powerful enough to detect effects of grammatical category, our results do not provide any support to the claim that grammatical category is a source of information for stress processing. In this respect, Italian differs from languages like English and Russian, where grammatical category was shown to play a role (Arciuli and Cupples, 2006; Jouravlev and Lupker, 2014). Most probably, the reason for this discrepancy is that in Italian, unlike those languages, grammatical categories do not have typical stress patterns – penultimate stress is the dominant pattern for all categories. Thus, although some differences exist, they are less informative to readers than more specific information (i.e., stress neighborhood), and distributional information at the level of grammatical categories may not be computed.

Experiment 2, on the other hand, showed that very specific distributional information coming from stress neighbors sharing morpho-syntactic properties with the target outweighs less specific information coming from stress neighborhood when the two contrast. This finding poses a challenge to current models of stress assignment and, in general, reading (e.g., Rastle and Coltheart, 2000; Ševa et al., 2009; Pagliuca and Monaghan, 2010; Perry et al., 2010). As no explicit information about morpho-syntactic properties is implemented in those models, they may fail to account for our results. Note that most models, however, successfully simulated stress typicality effects in English, suggesting that orthography, not grammatical category, has a causal role in driving stress assignment to nouns and verbs (see, e.g., Rastle and Coltheart, 2000; Perry et al., 2010). Since the correlation between orthographic units and grammatical category is so strong in English (see also Arciuli and Cupples, 2006, 2007), findings from different languages may help disentangle the two possible sources of information. Indeed, results from our Experiment 2 are independent of orthography, because if only orthographic units drove stress assignment, one could hardly explain the striking difference between the two experimental conditions (with and without context) in which the same orthographic endings were used. Thus, morpho-syntactic properties may be necessary for a complete account of stress assignment in reading aloud. It must be noted, however, that this conclusion relies on results with non-words; to fully account for how stress and morpho-syntactic properties interact in reading, future research might benefit from the investigation of real words too.

Our results are not conclusive as far as the *locus*/*loci* and mechanisms of the effects found are concerned. A viable hypothesis within a dual-route framework (Coltheart et al., 2001; Perry et al., 2010) is that the effect of morpho-syntactic properties on stress assignment to new words results from the activation of morpho-syntactically similar stress neighbors in the lexical route. In particular, we propose that the phonological lexicon is involved (Burani and Arduino, 2004). One may argue that the pattern we obtained arises at the orthographic level. However, since the non-words we used had no orthographic neighbors and participants had no time constraints, the obtained pattern of results is not likely to be the result of the erroneous activation of lexical units orthographically and morpho-syntactically similar to the target non-word. Alternatively, stress neighbors might be selectively activated by both the morpho-syntactic information processed within the lexical route and the phonological information already delivered in the phonological buffer by the sublexical route. In this view, feedback from the representation computed by the sublexical route would activate neighbors in the phonological lexicon, whereas morpho-syntactic information coming from the context word would restrict activation to those neighbors that are morpho-syntactically congruent with the target non-word. Thus, the context word would induce expectations concerning the morpho-syntactic properties of the target non-word, thereby enhancing the activation of words with those properties or similar to the representation previously computed by the sublexical route. More broadly, our study shows evidence of a syntax-to-phonology interaction. Our results would suggest that, at least to some extent, morpho-syntactic information rapidly contributes to the creation of the prosodic structure of the phrase, with information flowing from one level of processing to the other (Buxó-Lugo and Watson, 2016). The assumption of a tight relation between syntax and prosody is also in line with findings reported by the comprehension literature – which show that syntactic and prosodic information interact during sentence processing (e.g., Friederici, 2002). Although this hypothesis nicely fits our results, further research is needed to understand how and where information coming from morpho-syntactically similar stress neighbors is computed within the reading system and how it integrates syntactic and prosodic information.

In general, we believe that future research on stress processing and stress assignment will benefit from placing words and non-words in a sentence context, as the presence of a context may affect stress processing in non-obvious ways. In Experiment 1, we obtained the unexpected result that only inconsistent words were read faster when the context was available. Similarly, in Experiment 2, non-words with contrasting information coming from overall stress neighborhood and morpho-syntactically similar stress neighbors were found to be affected by the presence of context, whereas no such effect was expected for non-words for which the two sources of information agree. Taken together, results from Experiments 1 and 2 suggest that context does not affect stress assignment when a given stress pattern can be unambiguously assigned to the stimulus. This hypothesis, if true, would entail an interaction between contextual information and the mechanisms responsible for computing stress. More generally, we showed that the use of a context word affects reading aloud in important ways. To our knowledge, such a paradigm is almost new in naming studies. However, it yielded remarkable results for both words and non-words. Most importantly, it may serve as a way to go beyond single word reading and connect findings from the latter field with sentence reading – two areas of research that seldom communicate with each other.

## Author Contributions

GS designed and ran both experiments presented in the paper, as well as pilot studies. SS, SP, and CB supervised the experimental design and material selection. In addition, GS wrote the manuscript and SS performed the statistical analysis reported in the paper.

## Conflict of Interest Statement

The authors declare that the research was conducted in the absence of any commercial or financial relationships that could be construed as a potential conflict of interest.

## Footnotes

1 Capital letters indicate the stressed syllable.

2 There are a few exceptions to the phonological rule, such as *MANdorla* ‘almond’ and *POlizza* ‘[insurance] policy.’

3 http://www.pstnet.com

4 The same analysis was also run by including the length of the context word as a further predictor. In this way we could estimate to what extent the effect we reported could be accounted for by the variability of the context words we adopted. This new analysis showed the same pattern of results as the previous one.

## Appendix

### Experiment 1

#### Nouns with Consistent Stress Neighborhood

*Penultimate stress*: sottane, tisane, chimera, criniera, visiera, batteri, cimiteri, crateri, cometa, pineta, facchino, lavandino, listino, rullino, vaccino, granite, matite, pepite, graffiti, and quesiti *Antepenultimate stress*: maschere, sguattere, viscere, portici, salici, carotide, lapide, piramide, fossili, pugili, rettili, cresima, lacrima, bietole, donnole, ciondolo, discolo, pascolo, pendolo, and trespolo

#### Nouns with Inconsistent Stress Neighborhood

*Penultimate stress*: candela, parentela, bufere, galere, pancere, sombrero, torero, formica, mollica, vescica, attrici, narici, barile, porcile, vinile, braccioli, girasoli, lenzuoli, ravioli, and ghiaccioli *Antepenultimate stress*: bulgara, zingara, barbari, nettari, sigari, cardine, culmine, glutine, pettine, polline, redini, voragini, bibite, incognite, vincite, lasciti, moniti, diaspora, forfora, and tortora

#### Verbs with Consistent Stress Neighborhood

*Penultimate stress*: divago, propago, equipara, ripara, prepari, scompari, separi, abbina, declina, strofina, esplora, implora, pignora, affioro, divoro, perforo, configura, trascura, discuti, and incuti *Antepenultimate stress*: complica, implica, pizzica, applico, auspico, dimentico, interrogo, prorogo, brontola, dondola, rotola, brancoli, isoli, penzoli, pungoli, strangoli, postula, simula, specula, and ulula

#### Verbs with Inconsistent Stress Neighborhood

*Penultimate stress*: affida, confida, deride, incide, suddivide, compili, depili, infili, dirime, reprime, collimi, redimi, sopprimo, affogo, soggiogo, consola, immola, sorvola, accantono, and sprigiono *Antepenultimate stress*: agglomera, stempera, tollera, blateri, ponderi, superi, rumina, sanguina, stermina, dubita, evita, palpita, suscita, digito, eccito, imito, incito, precipito, amputi, and reputi

### Experiment 2

#### Non-words Presented as Nouns in the With-Context Condition

*Penultimate stress neighborhood*: cattrine, celdine, cuttine, daggine, dertine, falgine, fodine, gersine, gisine, mogine, norgine, ristine, tavine, trastine, vandine, and vorine *Antepenultimate stress neighborhood*: bintano, celpano, coddano, crentano, fepano, gespano, grelano, guvano, nabano, nipano, nircano, pralgano, sacano, sempano, trudano, vorbano, bostere, brelere, cofere, doltere, druggere, fagere, frellere, gardere, gistere, grentere, gutere, milgere, pimere, sandere, silere, togere, dafici, desici, furici, mardici, nassici, nuvici, repici, roltici, sompici, spalvici, sternici, tefici, tensici, tomprici, trunici, and vosici

#### Non-words Presented as Verbs in the With-Context Condition

*Penultimate stress neighborhood*: cebbera, cefera, corbera, dastera, gobera, grantera, lemera, lugera, pelgera, pitera, rogera, sertera, steldera, stogera, tabera, tandera, bigeri, caberi, colderi, daleri, deteri, dustreri, fasteri, gefferi, gelmeri, gileri, nogeri, pranteri, reberi, selgeri, spangeri, varderi, cobita, crombita, fostita, fupita, gerbita, getita, gipita, gossita, naprita, nicita, plamita, sfoccita, stecrita, trolita, tufita, and vuccita *Antepenultimate stress neighborhood*: bagide, catide, cepide, cettide, galmide, groside, magide, mostide, namide, pencide, pilide, ronide, spocide, tranide, vemide, and vuntide
